# FGF-2 inhibits contractile properties of valvular interstitial cell myofibroblasts encapsulated in 3D MMP-degradable hydrogels

**DOI:** 10.1063/1.5042430

**Published:** 2018-12-03

**Authors:** Andrea Gonzalez Rodriguez, Megan E. Schroeder, Cierra J. Walker, Kristi S. Anseth

**Affiliations:** 1 Department of Chemical and Biological Engineering, University of Colorado Boulder, Boulder, Colorado 80309, USA; 2 BioFrontiers Institute, University of Colorado Boulder, Boulder, Colorado 80309, USA; 3 Materials Science and Engineering Program, University of Colorado Boulder, Boulder, Colorado 80303, USA

## Abstract

Valvular interstitial cells (VICs) are responsible for the maintenance of the extracellular matrix in heart valve leaflets and, in response to injury, activate from a quiescent fibroblast to a wound healing myofibroblast phenotype. Under normal conditions, myofibroblast activation is transient, but the chronic presence of activated VICs can lead to valve diseases, such as fibrotic aortic valve stenosis, for which non-surgical treatments remain elusive. We monitored the porcine VIC response to exogenously delivered fibroblast growth factor 2 (FGF-2; 100 ng/ml), transforming growth factor beta 1 (TGF-β1; 5 ng/ml), or a combination of the two while cultured within 3D matrix metalloproteinase (MMP)-degradable 8-arm 40 kDa poly(ethylene glycol) hydrogels that mimic aspects of the aortic valve. Here, we aimed to investigate VIC myofibroblast activation and subsequent contraction or the reparative wound healing response. To this end, VIC morphology, proliferation, gene expression related to the myofibroblast phenotype [alpha smooth muscle actin (α-SMA) and connective tissue growth factor (CTGF)] and matrix remodeling [collagens (COL1A1 and COL3) and MMP1], and contraction assays were used to quantify the cell response. Treatment with FGF-2 resulted in increased cellular proliferation while reducing the myofibroblast phenotype, as seen by decreased expression of CTGF and α-SMA, and reduced contraction relative to untreated control, suggesting that FGF-2 encourages a reparative phenotype, even in the presence of TGF-β1. TGF-β1 treatment predictably led to an increased proportion of VICs exhibiting the myofibroblast phenotype, indicated by the presence of α-SMA, increased gene expression indicative of matrix remodeling, and bulk contraction of the hydrogels. Functional contraction assays and biomechanical analyses were performed on VIC encapsulated hydrogels and porcine aortic valve tissue explants to validate these findings.

## INTRODUCTION

Valvular heart disease is a major health problem throughout the world, and its prevalence is predicted to double before 2050.^1^ In the United States alone, fibrotic aortic valve stenosis (FAVS) affects 2.5% of the population,^2^ with valve replacement surgery remaining as the sole treatment. Valvular interstitial cells (VICs) are the primary cell population found in aortic heart valve leaflets. Under quiescent conditions, VICs predominantly exhibit a fibroblast phenotype but transition to an activated myofibroblast phenotype in response to injury. VIC myofibroblasts are characterized by the presence of alpha smooth muscle actin (α-SMA) stress fibers, contractility, and an increase of secretory properties and matrix remodeling.^3–6^ Under normal wound healing conditions, activated VIC myofibroblasts revert back to their quiescent fibroblast phenotype or undergo apoptosis^7,8^ after resolution of injury.^9^ However, when wound healing events become dysregulated, the activated myofibroblast phenotype can persist, and this persistence has been correlated with FAVS and valve calcification.^10^

Many early studies cultured VICs using traditional material substrates, such as tissue culture polystyrene (TCPS) or glass.^11–13^ While these studies provided important insights, the results are confounded by the fact that nearly 100% of VICs activate to myofibroblasts within 24–48 h of culture on surfaces with supraphysiological stiffnesses (∼1 GPa). Furthermore, VICs on two-dimensional (2D) surfaces cannot migrate, degrade, or contract the local environment in a manner that recapitulates the wound healing environment *in vivo*. Thus, more recent work has focused on understanding the development of the myofibroblast phenotype within three-dimensional (3D) biomaterial scaffolds. Implementing 3D cultures *in vitro* allows experimenters to control cell-matrix interactions, mechanosensing and force generation, and susceptibility of the matrix to degradation. In order to study the early stages of FAVS and misregulation of the wound healing process, a 3D environment is pivotal in the study of contractility development, a key property of persistently activated myofibroblasts.

Biochemical cues within the microenvironment are known to control the VIC phenotype. One of the most prominently studied cytokines that regulate the VIC myofibroblast transition is transforming growth factor beta 1 (TGF-β1).^14–18^ In fibroblasts, TGF-β1 induces α-SMA stress fiber formation, and these stress fibers play a critical role in wound contraction. Contractility is a key functional property for proper wound healing and scar formation, but aberrant myofibroblast activation promotes excessive extracellular matrix (ECM) contraction and remodeling, leading to fibrosis.

On the other hand, fibroblast growth factor 2 (FGF-2) reduces activation of the myofibroblast phenotype so that remodeling can occur without scarring.^19–21^ It is thought that FGF-2 preserves the fibroblast phenotype, replenishing cell populations in the area of injury via proliferation and migration for proper wound healing. It has yet to be established whether FGF-2 anti-fibrotic properties act through suppression of the fibrotic phenotype or via reparative healing processes.^22^ Cushing *et al.* showed that FGF-2 signals through mitogen-activated protein kinase (MAPK) cascades and inhibits TGF-β1-mediated activation via MAPK/extracellular regulated kinase (MEK-ERK)-dependent mechanisms to suppress the fibrotic phenotype.^23^ FGF-2 was reported to reduce α-SMA expression, collagen deposition, fibrotic contraction, and calcific nodule formation in aortic VIC cultures on tissue culture plasticware.^23^ Contrarily, Gotlieb *et al.* concluded that while FGF-2 promotes valve repair processes in mitral VICs, it also promotes the actin cytoskeletal organization orientation to support migrating cells required for wound healing.^24^ Although it is known that both TGF-β1 and FGF-2 are simultaneously present during the wound healing process, their antagonistic roles in FAVS are still not fully understood, and the specific contribution of FGF-2 to the reparative stage of aortic VICs in wound healing is still under debate.

Here, we aimed to measure the VIC response to the exogenous delivery of FGF-2 and TGF-β1 while encapsulated within a 3D matrix metalloproteinase (MMP)-degradable hydrogel tailored for VIC culture.^25^ To this end, we employed peptide-functionalized poly(ethylene glycol) (PEG) hydrogels in order to recapitulate the key properties of the biochemical and biomechanical valve leaflet microenvironment. The effect of FGF-2 and TGF-β1 treatment on encapsulated VIC myofibroblast activation and subsequent reparative or disease-like wound healing response was monitored. VIC phenotypic changes in response to FGF-2 and TGF-β1 were characterized at the gene and protein levels, as well as with functional contraction assays, and compared to healthy porcine valve tissue explants. To further understand FGF-2 and TGF-β1 antagonistic roles in VIC activation, the effect of combinatorial treatment on VIC α-SMA expression was studied.

## RESULTS

### Exogenous biochemical cues affect the morphology of encapsulated VICs

**FIG. 1.**
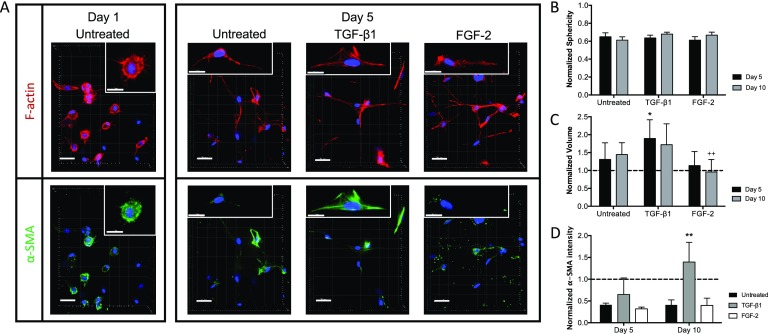
Morphological changes of encapsulated VICs in response to cytokine treatment. (a) Representative immunostaining images of VICs encapsulated within 3D MMP-degradable hydrogels at day 1 and day 5 of culture in untreated [10% fetal bovine serum (FBS) medium], TGF-β1 (5 ng/ml), and FGF-2 (100 ng/ml) conditions. Minimal spreading (F-actin, red) and diffuse α-SMA (green) are seen at day 1. By day 5, discrete F-actin stress fibers are seen in TGF-β1 and untreated conditions but absent from FGF-2 treated samples. Organized α-SMA fibers are present only in TGF-β1 treated hydrogels. Scale bars = 30 *μ*m. Inset scale bar = 10 *μ*m. (b) Sphericity decreased in all conditions after day 1 with no significant differences between treatments. Values normalized to day 1. (c) Cellular volume increased in untreated and TGF-β1 treated samples. TGF-β1 caused a 1.8-fold increase in cellular volume by day 5. Treatment with FGF-2 resulted in no change in cell volume and a lower cell volume than untreated controls. Values normalized to day 1 (dotted line). (d) Intensity of α-SMA relative to day 1 increased significantly for hydrogels treated with TGF-β1 in comparison to untreated and FGF-2 treated samples. Values normalized to day 1 (dotted line). **p < 0.01 and *p < 0.05. * indicates the significance of TGF-β1 treatment with respect to untreated conditions, and + indicates the significance of FGF-2 treatment with respect to untreated conditions.

VICs encapsulated within MMP-degradable matrices (i.e., 3D cultures) responded to cytokine treatments by changes in the morphology. This is observed qualitatively via immunostaining images demonstrating changes in cytoskeletal organization and stress fiber formation. Phenotypic changes were further quantified via analysis for α-SMA intensity, cellular volume, and sphericity using Imaris software. Hydrogels with VICs treated with either TGF-β1, FGF-2, or no treatment controls were fixed and stained for nuclei (blue), F-actin (red), and α-SMA (green) at days 1, 5 [Fig. 1(a)], and 10 (supplementary material Fig. 1). VICs responded to cytokines in a dose dependent manner (supplementary material Fig. 2), and concentrations of cytokines used in the following experiments were chosen for maximal cell activation, measured by α-SMA protein expression.

Initially (day 1 images), α-SMA staining was diffuse in VICs with no visible contractile fiber formation for all conditions, which also correlated with a lack of F-actin fiber organization. However, the F-actin structure varied greatly between conditions at day 5. In TGF-β1 treated VIC hydrogels, discrete, contractile F-actin fibers were observed, while F-actin in both FGF-2 treated and untreated VICs was disorganized with few observed contractile fibers. With respect to α-SMA stress fibers, both untreated and TGF-β1 treated VICs had increased organization of α-SMA fibers compared to day 1, while α-SMA remained primarily as diffuse or disorganized punctae in FGF-2 treated samples. The morphological differences between treatments are even more dramatic when observing samples in three dimensions (supplementary material, Videos 1–4). Sphericity decreased in all conditions after day 1; however, there were no significant differences between the treatments [Fig. 1(b)].

3D encapsulation also affected cellular volume [Fig. 1(c)]. With no exogenous treatment, VIC cellular volume increased at days 5 and 10 with respect to day 1, with no significant differences between these two later timepoints. TGF-β1 treatment also caused an increase in volume with an approximate 1.8-fold increase in cell volume after 5 days. This TGF-β1-induced volume increase after 5 days was significantly higher than the untreated condition. Interestingly, FGF-2 did not cause any significant change in cell volume after either 5 or 10 days and prevented any increase in cellular volume, indicated by the significantly lower cell volume compared to untreated control at 10 days.

At the initial time point (day 1), VICs expressed a high level of α-SMA. By 5 days, both untreated and FGF-2 treated VICs decreased their α-SMA expression levels, while TGF-β1 treated VICs remained elevated [Fig. 1(d)]. After 10 days of treatment, VICs treated with TGF-β1 expressed significantly more α-SMA as measured by intensity compared to either untreated VICs or FGF-2 treated VICs. While the myofibroblast phenotype is traditionally characterized by the presence of organized α-SMA stress fibers, the morphological changes observed in the images after treatment with FGF-2 warranted deeper exploration of the cellular phenotype at the genetic level.

### FGF-2 but not TGF-β1 increases proliferation in encapsulated VICs

**FIG. 2.**
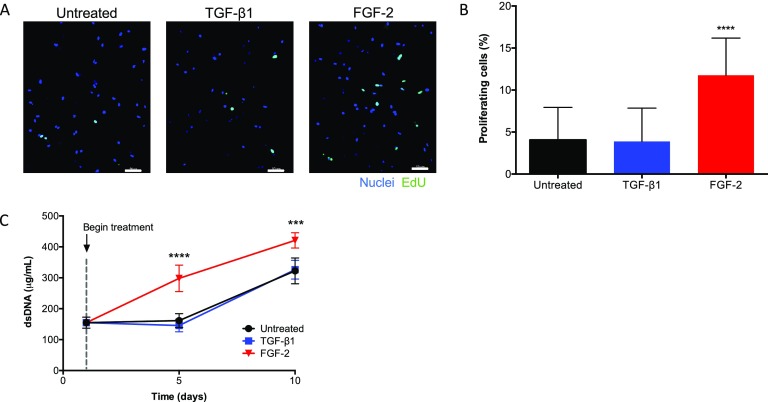
VICs proliferate within 3D MMP-degradable hydrogels in response to cytokine treatment. (a) Representative images for EdU staining of MMP-degradable encapsulated VICs after culturing for 5 days in untreated (10% FBS medium), TGF-β1 (5 ng/ml), or FGF-2 (100 ng/ml) conditions. FGF-2 treatment increased the number of EdU positive cells compared to TGF-β1 and untreated controls. Nuclei (blue) and EdU (green). Scale bars = 50 *μ*m. (b) Quantification of the percent proliferating cells with respect to treatment. FGF-2 treatment increased the number of EdU positive cells to 11.7% ± 4.4% relative to untreated controls. (c) Quantification of the dsDNA content over time for VIC-laden hydrogel with respect to treatment. Treatment with FGF-2 resulted in significantly higher dsDNA after day 5 and day 10 culture, compared to either TGF-β1 or untreated conditions. ****p < 0.0001 and ***p < 0.001.

Proliferation of VICs encapsulated within MMP-degradable hydrogels was assessed over time. For untreated conditions, 4.1% ± 3.9% of VICs were EdU positive at day 5 [Figs. 2(a) and 2(b)]. The double-stranded DNA (dsDNA) content in untreated controls at day 1 was 155 ± 16 mg/ml and increased over 10 days to 322 ± 30 mg/ml [Fig. 2(c)]. Samples treated with TGF-β1 were not significantly different from untreated controls in either percent of proliferating VICs or dsDNA content. Hydrogels treated with FGF-2 had more EdU-positive cells (day 5 = 11.7% ± 4.4%) relative to untreated samples and increased cellularity with time (dsDNA =  298 ± 47 mg/ml at day 5 and 421 ± 24 mg/ml at day 10). Over 10 days, FGF-2 had a significant effect on VIC proliferation and final cell content within the hydrogels, while TGF-β1 treatment was similar to untreated control.

### 3D-encapsulated VIC gene expression response to TGF-β1 and FGF-2

**FIG. 3.**
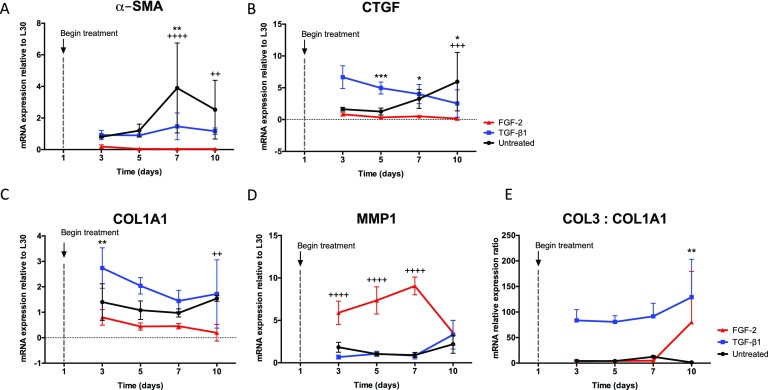
mRNA levels of myofibroblast markers and matrix remodeling markers in response to TGF-β1 and FGF-2 treatment. [(a), (b)] Expression of myofibroblast markers in VIC-laden hydrogels relative to L30. Both α-SMA (a) and CTGF (b) were quantified in response to TGF-β1 (5 ng/ml) and FGF-2 (100 ng/ml) treatment and compared to untreated control at days 3, 5, 7, and 10. FGF-2 treatment suppressed the expression of myofibroblast genes, as seen via downregulation of both α-SMA and CTGF with respect to untreated control. Treatment with TGF-β1 resulted in similar mRNA levels of α-SMA at early time points compared to untreated control but was downregulated at later time points. CTGF mRNA expression in TGF-β1 samples was upregulated at day 3 with respect to untreated control but decreased over time. VICs in the untreated condition had an opposite trend with increasing CTGF expression with time, reaching a higher fold expression than TGF-β1 treated VICs at day 10. [(c) and (d)] Expression of matrix associated genes in VICs relative to L30. VIC expression of (c) COL1A1 and (d) MMP1 was measured in response to TGF-β1 (5 ng/ml) and FGF-2 (100 ng/ml) treatment and compared to untreated control at days 3, 5, 7, and 10. FGF-2 treatment reduced expression of COL1A1 but significantly upregulated MMP1 expression at earlier time points relative to untreated control, indicative of matrix remodeling in response to treatment. TGF-β1 treatment led to an initial peak in COL1A1 expression that decreased with time, while MMP1 expression remained relatively low and constant. There was little change in either COL1A1 or MMP1 expression in untreated conditions over time. (e) COL3 to COL1A1 mRNA ratio with TGF-β1 treatment resulted in significant upregulation at all time points, whereas FGF-2 treatment resulted in similar values to those of untreated samples. ****p < 0.0001, ***p < 0.001, **p < 0.01, and *p < 0.05. * indicates the significance of TGF-β1 treatment with respect to untreated conditions, and + indicates the significance of FGF-2 treatment with respect to untreated conditions.

Gene expression of myofibroblast markers [Figs. 3(a) and 3(b)] was analyzed to assess the VIC response to TGF-β1 and FGF-2 treatment over time when cultured in a 3D hydrogel microenvironment. In general, FGF-2 suppressed the myofibroblast phenotype, as observed by downregulation of both α-SMA and connective tissue growth factor (CTGF) expression compared to untreated samples. TGF-β1 treated samples expressed similar levels of α-SMA relative to untreated samples at early time points but were downregulated after day 5. In contrast, CTGF expression with TGF-β1 treatment was significantly upregulated on day 3 and decreased over time, whereas untreated samples followed the opposite trend, with expression elevated 5-fold by day 10. These data indicate that the untreated condition had a delayed myofibroblast response compared to TGF-β1 treatments, which showed upregulated myofibroblast markers at earlier time points.

The expression of matrix remodeling genes [Figs. 3(c) and 3(d)] was measured to assess changes in VIC secretory properties upon long-term treatment with TGF-β1 and FGF-2. Samples treated with FGF-2 showed low levels of collagen type I (COL1A1) expression at all time points. However, there was a significant increase in MMP1 expression (3-fold) relative to untreated samples by day 3. TGF-β1 treatment resulted in a significant upregulation of COL1A1 expression (2-fold) compared to untreated samples at day 3 but followed a decreasing trend over time. MMP1 expression levels were not significantly different from those of samples with no exogenous treatment. The collagen type III to collagen type I (COL3:COL1A1) mRNA ratio was calculated as an additional measurement of the VIC fibrotic phenotype because this ratio increases at early time points in wound healing processes^26^ [Fig. 3(e)]. TGF-β1 treatment resulted in a significant increase in the COL3:COL1A1 ratio relative to untreated by day 10, whereas FGF-2 treatment resulted in similar values to those of untreated samples.

### VICs respond similarly to TGF-β1 treatment in hydrogel microenvironments and valve tissue explants

**FIG. 4.**
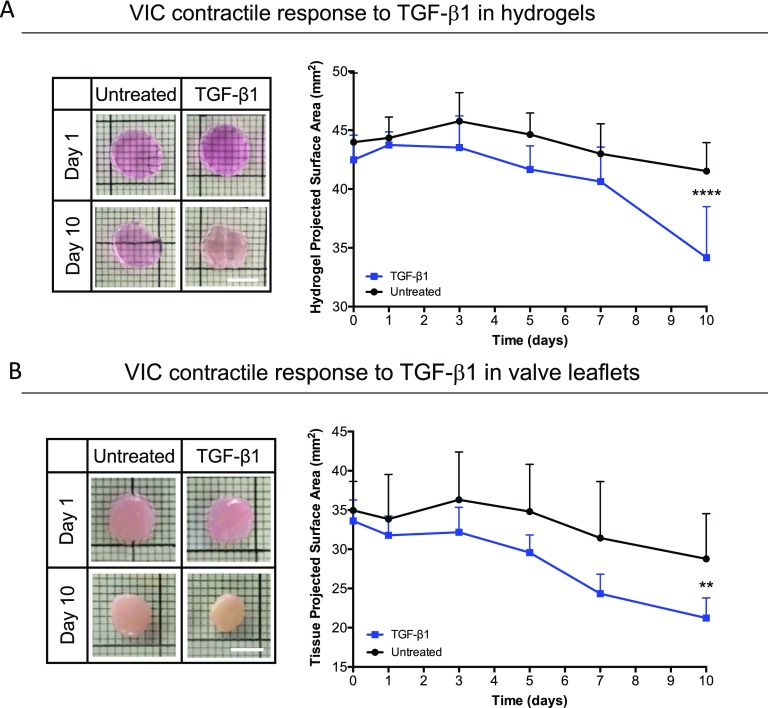
Functional contraction observed in response to treatment with TGF-β1 to VICs cultured in hydrogels versus porcine aortic valve tissue explants. (a) TGF-β1 treatment to VIC-laden MMP-degradable hydrogels resulted in significant contraction (19.5% ± 9.7% decrease in the gel area between day 0 and day 10) as measured via the projected surface area, relative to a 4.4% ± 11.4% decrease in the surface area of untreated control hydrogels. Images demonstrate bulk contraction and opacity due to densification. (b) Valve tissue explants contract with TGF-β1 treatment over 10 days, resulting in a decrease of 36.5% ± 9.1% in the projected surface, relative to 17.9% ± 12.7% reduction in the area for untreated tissue samples. Images demonstrate bulk contraction of tissue explants. Scale bars = 5 mm. ****p < 0.0001 and **p < 0.01. * indicates the significance of TGF-β1 treatment with respect to untreated conditions.

Differences in functional contraction were observed with treatment of exogenous TGF-β1 relative to untreated controls in both VIC-laden MMP-degradable hydrogels and porcine aortic valve tissue explants (Fig. 4). Cell-laden hydrogels were treated with TGF-β1 for 10 days, resulting in significant matrix contraction (19.5% ± 9.7% decrease in the gel area between day 0 and day 10) [Fig. 4(a)] and an increase in hydrogel opacity (supplementary material Fig. 3). Untreated samples exhibited a slight size reduction (4.4% ± 11.4% decrease) but were not statistically different from an acellular control (supplementary material Fig. 4). Tissue explants were cultured for 10 days and resulted in a significant 36.5% ± 9.1% reduction in the projected surface area with TGF-β1 treatment, relative to 17.9% ± 12.7% reduction in the area for the untreated controls [Fig. 4(b)].

### FGF-2 treatment inhibits VIC contraction and α-SMA protein expression in hydrogel cultures

**FIG. 5.**
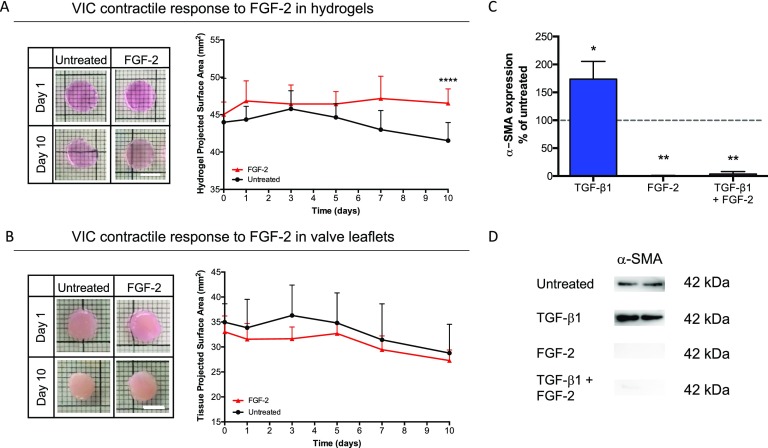
FGF-2 treatment inhibits VIC contraction and α-SMA protein expression in hydrogels. (a) FGF-2 treatment to VIC-laden MMP-degradable hydrogels resulted in an increase in the projected surface area (3.3% ± 2.6%) over time, which was statistically different from the untreated condition (4.4% ± 11.4% reduction in the area). Images demonstrate the lack of contraction between conditions. (b) Tissue explant contraction in response to FGF-2 treatment (17.2% ± 4.8% reduction in the area) was not statistically different from the untreated controls (17.9% ± 12.7% reduction in the area). Images demonstrate lack of bulk contraction of tissue explants. (c) Percentage α-SMA protein expression relative to untreated conditions (dotted line). TGF-β1 treatment significantly increased protein expression (73.7% ± 31.7%), whereas FGF-2 resulted in a significant decrease (99.6% ± 0.6%). Combinatorial treatment of exogenous cues resulted in significantly lower α-SMA protein expression (96.3% ± 4.3%), similar to that of FGF-2 alone. (d) Representative western blot used for analysis, all lanes from the same blot. Scale bars = 5 mm. ****p < 0.0001, **p < 0.01, and *p < 0.05. * indicates the significance of treatment with respect to untreated conditions.

Contraction is inhibited by treatment with exogenous FGF-2 for 10 days for VIC-laden MMP-degradable hydrogels (Fig. 5). Cell-laden hydrogels were either untreated or treated with FGF-2 for 10 days [Fig. 5(a)]. Quantification between day 0 and day 10 of the projected surface area of hydrogels in response to FGF-2 treatment resulted in a slight increase in the projected surface area (3.3% ± 2.6%) over time, which was statistically different from the untreated condition (4.4% ± 11.4% area reduction), but not acellular controls (supplementary material Fig. 4). FGF-2 treatment also resulted in increased hydrogel opacity at day 10 (supplementary material Fig. 3). Tissue explants were cultured similarly to hydrogels and resulted in a reduction of 17.2% ± 4.8% for FGF-2 treatment and 17.9% ± 12.7% for untreated samples, which is not statistically different [Fig. 5(b)].

In order to study the combinatorial effects of FGF-2 and TGF-β1 treatment, α-SMA protein expression was evaluated for cell-laden hydrogels cultured for 10 days with FGF-2, TGF-β1, or a combination of both exogenous cues [Figs. 5(c) and 5(d)]. Quantification resulted in significantly higher expression of α-SMA (73.7% ± 31.7% increase) with TGF-β1 treatment relative to untreated samples, while FGF-2 treatment significantly reduced α-SMA protein expression (99.6% ± 0.6% decrease). Combinatorial treatment (TGF-β1 and FGF-2) resulted in a significant decrease in α-SMA with respect to untreated samples (96.3% ± 4.3% decrease), demonstrating a potential protective effect of FGF-2 against VIC activation.

### Exogenous cytokine treatment causes similar responses in contraction and mechanical properties in both hydrogels and tissues

**FIG. 6.**
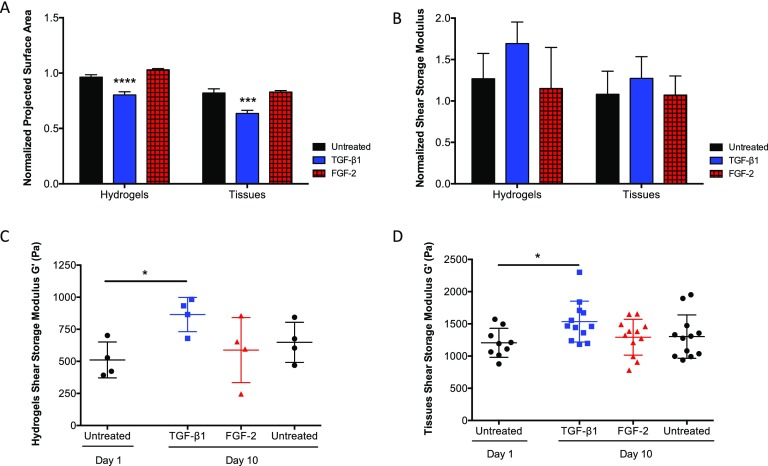
Hydrogel results validated by similar trends in porcine aortic valve tissue explants. (a) Normalized projected surface area (day 10/day 0) contraction trends with respect to treatment conditions are conserved between MMP-degradable hydrogels and tissue explants. Treatment with TGF-β1 resulted in significant contraction in both culture constructs. (b) Normalized shear storage modulus (day 10/day 1) trends are conserved between hydrogels and tissue explants, where treatment with TGF-β1 resulted in increased stiffness. (c) Hydrogel shear storage modulus measured for samples at day 1 vs. day 10 plus treatment conditions. TGF-β1 treatment resulted in a 1.7-fold increase in G′ between day 1 and day 10. (d) Tissue explant shear storage modulus measured for samples at day 1 vs. day 10 plus treatment conditions. TGF-β1 treatment resulted in a 1.3-fold increase in G′ between day 1 and day 10. ****p < 0.0001, ***p < 0.001, and *p < 0.05.

The response to exogenously delivered factors (TGF-β1 and FGF-2) was compared for both VICs encapsulated in MMP-degradable hydrogels and VICs in porcine aortic valve explants (Fig. 6). The normalized projected surface area indicated that both hydrogel cultures and tissue explants contract in a similar manner with treatment of TGF-β1, and the trend is conserved between culture constructs [Fig. 6(a)]. Furthermore, the trends in mechanical properties changed over time between untreated samples and either TGF-β1 or FGF-2 treatment, as quantified by the shear storage modulus, and are conserved between hydrogels and tissue explants, although not statistically different between conditions [Fig. 6(b)]. The shear storage modulus was measured and compared between day 1 and day 10 samples for both hydrogels and tissues and for all treatment conditions [Figs. 6(c) and 6(d)]. The shear storage modulus increased with treatment of TGF-β1 relative to untreated by 70% for hydrogels and 30% for tissues which is likely due to the increased complexity of the leaflet tissue (e.g., hierarchical tissue organization, fibrillary nature, and stiffening) relative to the amorphous hydrogels. While the magnitudes of the functional output of VIC contraction and change in matrix shear storage modulus are varied between tissue and the hydrogel microenvironment, the trends are conserved, thereby validating this culture system as an *in vitro* platform that mimics aspects of the native tissue.

## DISCUSSION

Early causes of fibrotic aortic valve stenosis remain enigmatic, yet identifying how VICs respond to injury, especially during early stages of disease, could prove useful in future studies for identifying drug therapeutics to mitigate the effects of this disease. The effects of pro-inflammatory cytokines and growth factors on VICs cultured on 2D substrates have been well studied, but how VICs respond to biochemical signals in a more physiologically relevant 3D microenvironment remains an important link to improving the understanding of the VIC's role in FAVS. Here, we encapsulated porcine aortic valve cells within a 3D soft hydrogel formulation, which maintains VIC quiescence and allows them to degrade and interact with their matrix.

In these experiments, we aimed to investigate the reparative role of FGF-2 treatment to VICs encapsulated within a 3D environment which more closely mimics the native environment. Culturing cells in 3D allows for bulk contraction, which is a key component of the wound healing response of activated myofibroblasts, and is an advantage over studying the VIC response on traditional 2D systems. FGF-2 is a known mitogen, causing fibroblasts to proliferate, and is involved with fibroblast-mediated wound healing. Because fibroblast proliferation is associated with early stages of fibrosis, FGF signaling has been implicated to play a pro-fibrotic role.^27^ However, recent studies have demonstrated that myofibroblast activation can be attenuated with FGF-2 treatment. For example, genetic knockdown of FGF-2 prevented proper tissue recovery in a lung injury mouse model, indicating a protective role in wound healing.^28^ We sought to determine how FGF-2 might affect VICs in a physiologically relevant mechanical hydrogel microenvironment.

We first investigated how exogenous cytokine treatment affected the cell morphology and myofibroblast activation in 3D. The concentrations of biochemical cues were chosen based on maximal VIC activation, measured by the highest α-SMA protein expression (supplementary material Fig. 2), and are within the realm of human circulating concentrations.^8,14,23,29–31^ To study combinatorial effects, these cytokines were then combined at the selected concentrations above. As expected, we observed that TGF-β1 treatment resulted in increased levels of myofibroblast activation, as indicated by both qualitative and quantitative analyses of α-SMA expression in comparison to untreated samples (Figs. 1, 3, and 5). Additionally, TGF-β1 treatment caused an increase in cell volume (Fig. 1). We observed that FGF-2 treatment caused no significant changes in quantitative myofibroblast activation levels, as measured by normalized α-SMA intensity, but did result in significantly decreased protein expression of α-SMA (Figs. 1 and 5) and interesting alterations in the cell morphology (Fig. 1). This discrepancy is likely due to limitations with immunostaining quantification in 3D culture systems. Qualitatively, VICs appeared to develop spindle-like protrusions in response to FGF-2 treatment. Additionally, FGF-2 prevented an increase in cellular volume associated with longer encapsulation times as seen between day 1 and day 10 untreated conditions. Together, these data suggest that FGF-2 is not pro-fibrotic like TGF-β1 and may even provide a protective effect.

Next, we examined how cytokine treatment affected the proliferative phenotype of VICs. TGF-β1 had no measurable increase in VIC proliferation over 5 days (Fig. 2). This result is different from those reported by Sadeghi *et al.* who observed that TGF-β1 (2 ng/ml) led to increased cardiac fibroblast proliferation,^32^ while others have shown inhibition of VIC proliferation at various TGF-β1 treatment concentrations (0.5–5 ng/ml).^33^ Thus, TGF-β1's influence on VIC proliferation is not definitively known, and microenvironmental conditions may further influence the VIC response. Meanwhile, there was a significant increase in the number of proliferating cells, resulting in increased cell density (i.e., total dsDNA content), with FGF-2 treatment. This result indicates that FGF-2 may provide a protective effect through increased proliferation.

Genes related to myofibroblast and matrix remodeling markers were studied over time in VIC-laden hydrogels (Fig. 3). With TGF-β1 treatment, both α-SMA and CTGF mRNA levels indicated a relatively rapid myofibroblast transition, as one might expect. We monitored changes in both MMP1 and COL1A1 gene expression in VICs, as an indicator of their matrix synthesis and remodeling properties. Upregulation of COL1A1, a precursor of type I collagen, was observed with TGF-β1 treatment, and this upregulation was 2-fold higher compared to untreated control at day 3. The increased COL1A1 expression in VICs is associated with higher collagen type I deposition, which is necessary for proper wound healing. However, excessive collagen deposition is also associated with fibrosis, as excess and disorganized collagen is correlated with increased leaflet stiffness and persistent myofibroblast activation.^34–36^ Interestingly, VICs treated with TGF-β1 did not have elevated MMP1 gene expression, so in combination with the increased COL1A1 expression, VICs are likely actively depositing matrix. With FGF-2 treatment, the myofibroblast markers α-SMA and CTGF remained low compared to TGF-β1 treated and untreated VICs, again suggesting a protective role of FGF-2 in the fibrotic response. On the other hand, MMP1 mRNA levels were significantly upregulated in VICs, and this observation may indicate FGF-2 biases VICs towards remodeling and degrading their microenvironment. This notion is consistent with the observed increase in VIC proliferation with FGF-2 treatment, as the rate of cellular division in these gels is limited by the rate of matrix degradation.

The collagen type III to collagen type I ratio was calculated as an additional measurement of fibrosis and wound healing [Fig. 3(d)]. An alteration in the COL3:COL1A1 ratio has been observed in a number of fibrotic diseases, including aortic stenosis.^37^ Interestingly, at late time points of disease, there is an overall decrease in the COL3:COL1A1 ratio.^37^ However, at early time points of fibrotic disease progression, there is an increase in the COL3:COL1A1 ratio, suggesting that upregulation of COL3 may play a role in initial stages of fibrosis development.^38^ In support of this argument, we saw an increase in the COL3:COL1A1 ratio at nearly all time points with TGF-β1 treatment, significantly upregulated by day 10. FGF-2, on the other hand, showed no significant differences compared to untreated VICs. These data support our hypothesis that FGF-2 does not contribute to fibrosis initiation or development, while TGF-β1 does.

Contractility is one of the key functional properties of myofibroblasts, which plays a major role during wound healing and when misregulated, scarring. In this work, we used a functional contraction assay to investigate the ability of VICs to coordinate and contract their 3D bulk microenvironment in response to exogenous treatment with TGF-β1 or FGF-2. Figure 4 shows significant VIC-mediated hydrogel contraction upon treatment with TGF-β1. These results confirm a coordinated cell response in 3D in order to macroscopically remodel their environment and contract the hydrogel. Furthermore, the increase in the projected surface area was measured in a post-kill experiment to confirm that the observed contraction is due to active contractility of the cells (supplementary material Fig. 5). Treatment with FGF-2 inhibited hydrogel contraction [Fig. 5(a)]. This lack of contraction, along with the absence of F-actin fibers and gene expression indicating higher matrix remodeling, supports the conclusion that FGF-2 treatment promotes a VIC phenotype focused on tissue repair. Furthermore, the downregulation of myofibroblast related gene expression indicates a fibroblast phenotype.

In healthy valve tissue, it has been proposed that autocrine and paracrine expression of FGF-2 may be a mechanism for cells to maintain the quiescent VIC phenotype.^39^ During wound healing, it is likely important for VICs to self-regulate, as there is increased production of TGF-β1 and other pro-fibrotic factors that can lead to valve fibrosis. Investigating the interplay of exogenous factors is important to understand normal valve function and thus have a better indication of what mechanisms may be disrupted in disease. The combinatorial effect of TGF-β1 and FGF-2 on VIC activation is not well established, with conflicting results of FGF-2 preventing TGF-β1-mediated Smad expression^23^ or promoting TGF-β1/Smad signaling.^12^ Our results suggest that FGF-2 almost completely overrides the pro-fibrotic effect of TGF-β1 [Fig. 5(c)]. This result suggests that if normal FGF-2 production is reduced or absent, fibrosis may follow. Future studies focusing on the combinatorial effects of pro- and anti-fibrotic biochemical cues would benefit from modifying the timelines of treatment (i.e., addition and removal of biochemical cues), correlated with different wound healing stages, as well as investigating the temporal effect on VIC phenotype reversibility.

Contractility trends between hydrogel-encapsulated VICs and tissue explants showed that cells respond in a similar manner in both matrices (Fig. 6). Furthermore, mechanical characterization of the *ex vivo* and *in vitro* VIC microenvironments showed an increase in shear storage modulus when treated with TGF-β1 for 10 days in both hydrogels and tissue explants. FGF-2 treatment did not inhibit tissue contraction as seen in hydrogels matrices, with results similar to untreated samples. The discrepancies observed between hydrogel and tissue explant results can likely be attributed to the increased complexity of the tissue structure over the amorphous hydrogels that lack fibrillary ECM structures, as well as differences in cell proliferation and remodeling in response to FGF-2 treatment. Overall, these results support the relevance of studying VICs in 3D matrices, rendering this hydrogel system as a useful tool for investigating defined hypotheses that are impractical to conduct *in vivo* or with leaflet culture studies. Additionally, these results support the hypothesis that FGF-2 plays both a regenerative and an anti-fibrotic role in aortic valve fibrosis through increased matrix remodeling, proliferation, and inhibition of contraction and pro-fibrotic markers.

## METHODS

### Valvular interstitial cell isolation and expansion

Porcine hearts were obtained within 24 h of slaughter from Hormel Foods Corporation (Austin, MN, USA). Aortic valve leaflets (the half most distal from root, encompassing all three layers) were excised, washed with Earle's Balanced Salt Solution (Sigma-Aldrich), and digested with Collagenase Type II solution (Worthington Biochemical Corporation). Samples were then vortexed and filtered with a 100 *μ*m cell strainer to remove any remaining tissue. Isolated VICs were then resuspended in media M199 (ThermoFisher Scientific) supplemented with 15% fetal bovine serum (FBS, ThermoFisher Scientific) and plated for expansion.

### Cell culture

Encapsulated cells were kept in 1% FBS M199 media overnight (day 0). On day 1, gels were cultured in 700 *μ*l of either 10% FBS M199 untreated, treated with 5 ng/ml porcine TGF-β1 (R&D Systems), or 100 ng/ml of FGF-2 (Millipore Sigma). The culture medium was refreshed every other day.

Tissue biopsies of aortic valve leaflets were obtained with a 6 mm biopsy punch, farthest from the cusp-edge of the sample. Once excised, tissue biopsies were kept in 10% FBS M199 media overnight (day 0), and starting on day 1, they were cultured with the same conditions described for encapsulated cells.

### Materials synthesis, cell encapsulation, and characterization

Eight-arm 40 kDa poly(ethylene glycol) (PEG) was functionalized with norbornene as previously described.^40^ The extent of end-group functionalization was confirmed by proton nuclear magnetic resonance imaging to be ∼88%; 1H nuclear magnetic resonance (NMR) (400 MHz, CDCl3, δ): 6.25–5.95 (m, 2H) and 3.85–3.25 (m, PEG, 260H).

VICs were encapsulated at 10 × 10^6^ cells/mL within an MMP-degradable, PEG hydrogel via photo-initiated thiol-ene polymerization. The polymer precursor containing 8-arm 40 kDa PEG norbornene ([enes] = 6 mM), photoinitiator lithium phenyl-2,4,6-trimethylbenzoylphosphinate ([LAP] = 1.7 mM), CRGDS adhesive peptide ([thiols] = 1 mM, Bachem), and peptide crosslinker KCGPQG↓IWGQCK ([thiols] = 3.34 mM, Bachem). This polymer precursor was then mixed with cell solution and polymerized for 3 min under 365 nm 2.5 mW/cm^2^ light to form 1 mm  ×  6 mm hydrogel discs.

Swollen hydrogel and tissue mechanical properties were characterized via a DHR3 rheometer (TA instruments). Frequency and strain sweeps were conducted at 37 °C using an 8 mm Peltier Plate tool covered with adhesive 600/P1200 sandpaper to prevent slippage.

### Cellular phenotype characterization [quantitative polymerase chain reaction (qPCR), immunostaining, EdU Assay, PicoGreen Assay, and western blots]

#### RNA isolation and quantitative real-time polymerase chain reaction (RT-qPCR) assessment of gene expression

Total RNA was isolated from hydrogels using TRI Reagent (Sigma-Aldrich). In brief, gels were digested using 2 mg/ml Collagenase Type I (Worthington Biochemical Corporation) to collect cells that were then lysed in 500 *μ*l of TRI Reagent. RNA was isolated using a chloroform extraction and excess 2-propanol (Sigma-Aldrich) to precipitate it, and pellets were washed and resuspended in RNAse free water (Sigma-Aldrich). The RNA concentration and quality were assessed with a ND-1000 Nanodrop Spectrophotometer. For RT-qPCR, cDNA was synthesized with the iScript Reverse Transcription Supermix kit (Bio-Rad) and an Eppendorf Mastercycler. Relative mRNA expression levels were measured using SYBR Green reagents (Bio-Rad) using an iCycler machine (Bio-Rad), normalizing to the housekeeping gene (ribosomal protein L30). Custom primers are presented in Table 1 in the supplemental material.

#### Immunostaining for α-SMA, actin filaments, and nuclei

Encapsulated cells were fixed for 30 min with 10% formalin (Sigma-Aldrich), permeabilized with 0.05% Tween20 (Sigma-Aldrich) in phosphate-buffered saline (PBS) (PBST), and blocked with 1% bovine serum albumin (BSA, Sigma-Aldrich) in PBST. Primary anti-mouse alpha smooth muscle actin (α-SMA, Abcam) was added 1:1000 overnight at 4 °C in blocking solution. Samples were washed with PBST, and secondary antibody goat-anti-mouse AlexaFluor 488 (Abcam, 1:300) in 1% BSA in PBST was added overnight at 4 °C. F-actin filaments were labeled with Rhodamine Phalloidin (ThermoFisher Scientific, 1:300) and the cell nucleus labeled with 4′,6-Diamidino-2-Phenylindole (DAPI, ThermoFisher Scientific, 1:1000).

#### Proliferation EdU and PicoGreen assays

The Click-iT EdU Alexa Fluor 488 Imaging Kit (ThermoFisher Scientific) was used with a 17 h pulse of 5-ethynyl-2′-deoxyuridine (EdU) at 10 *μ*M. Samples were then immunostained for DAPI as previously described. The Quant-iT PicoGreen Assay kit (ThermoFisher) was used to quantify the dsDNA content according to the manufacturer's instructions for the Quant-iT PicoGreen Assay Kit (ThermoFisher).

#### Western blots

Chemiluminescence western blot techniques were used to assess the α-SMA protein relative to the total protein (Ponceau stain). Polyvinylidene difluoride (PVDF) membranes (Bio-Rad) were used and probed for the primary α-SMA antibody (Abcam, 1:1000) diluted in blocking solution (5% skim milk) at 4 °C. Membranes were incubated with a secondary goat-anti-mouse horseradish peroxidase conjugated antibody (Jackson ImmunoResearch, 1:5000) for 1 h at RT. The chemiluminescence signal was detected using Pierce enhanced chemiluminescence (ECL) Plus solution (ThermoFisher Scientific) and an ImageQuant LAS 4000 detector.

### Contraction assays

Both gels and tissue biopsies were placed on a microscope slide on top of 10 mm graph paper and imaged on days 0, 1, 3, 5, 7, and 10. Images were then used to calculate the projected surface area to assess contraction over time.

### Microscopy and analysis

Images were acquired via a Nikon Ti-E Spinning Disc Confocal. Image analysis was performed with either Microscopy Imaging Analysis Software (Bitplane) or FIJI.^41^

### Statistical analysis

Data are presented as mean ± standard deviation (SD) with a minimum of three biological replicates with at least two technical replicates for all studies. Western blot data are presented with a minimum of two biological replicates. Data were analyzed by analysis of variance (ANOVA) using SPSS (IBM v.24) or Graph pad™ (Prism).

### Ethics

No ethics approval was required for this study.

## SUPPLEMENTARY MATERIAL

See supplementary material for more detailed methods and additional experimental figures.
